# Antifungal stewardship in a tertiary care paediatric hospital: the PROAFUNGI study

**DOI:** 10.1186/s12879-021-05774-9

**Published:** 2021-01-22

**Authors:** Natalia Mendoza-Palomar, Beatriz Garcia-Palop, Susana Melendo, Maria Teresa Martín, Berta Renedo-Miró, Pere Soler-Palacin, Aurora Fernández-Polo

**Affiliations:** 1 Pediatric Infectious Diseases and Inmunodeficiencies Unit, Hospital Universitari Vall d’Hebron, Institut de Recerca Vall d’Hebron, Universitat Autònoma de Barcelona, Passeig de la Vall d’Hebron, 119-129, 08035 Barcelona, Spain; 2 Pharmacy Department, Hospital Universitari Vall d’Hebron, Institut de Recerca Vall d’Hebron, Barcelona, Spain; 3 Microbiology Department, Hospital Universitari Vall d’Hebron, Institut de Recerca Vall d’Hebron, Universitat Autònoma de Barcelona, Barcelona, Spain

**Keywords:** Antifungal therapy, Paediatrics, Antimicrobial management

## Abstract

**Background:**

The increasing use of antifungal drugs (AF) in children and the concern for related adverse events and costs has led to the development of specific AF stewardship programmes (AFS). Studies in adult patients have shown improvements in AF prescription and usage after implementation, but paediatric data are scant. The aim of this PROAFUNGI study was to describe the use and appropriateness of AF in a high complexity paediatric centre.

**Methods:**

Observational, prospective, single-centre, modified point-prevalence study (11 surveys, July–October 2018), including paediatric (< 18 years) patients receiving at least one systemic AF. Prescriptions were evaluated by the AFS team.

**Results:**

The study included 119 prescriptions in 55 patients (53% males, median age 8.7 years [IQR 2.4–13.8]). The main underlying condition was cancer (45.5% of patients; HSCT in 60% of them); and the first indication for AF was prophylaxis (75 prescriptions, 63.2%). Liposomal amphotericin B was used most commonly (46% prescriptions), mainly as prophylaxis (75%). Among the 219 evaluations, 195 (89%) were considered optimal. The reason for non-optimal prescriptions was mostly lack of indication (14/24), especially in critical patients with ventricular assist devices. The use of AF without paediatric approval accounted for 8/24 inappropriate prescriptions.

**Conclusions:**

A high rate of AF appropriateness was found for the children’s hospital as a whole, in relation with a well-established AFS. Nonetheless, the identification of specific areas of improvement should guide future actions of the AFS team, which will focus mainly on prophylaxis in critically ill patients receiving circulatory assistance and the use of non-approved drugs in children.

## Background

In recent years, the increase in the number of immunosuppressed children at risk of invasive fungal infection (IFI) has led to a higher use of both prophylactic and therapeutic antifungal drugs (AF) in this population [1]. In these patients, AF prescription may be challenging due to unspecific clinical presentation, lower performance of diagnostic tests, variable pharmacokinetics related to maturation changes and lack of clinical trials in children [1–3].

Unnecessary AF use entails substantial risk of toxicity and interactions, along with higher drug resistances and significant costs [3]. In the last 5 years, AF drugs accounted for around one-third of antimicrobial usage (13.79% of all days of therapy [DOT] per 100 patient-days [PD]) but 75% (€638,403) of total annual anti-infective expenditure in our hospital (unpublished data).

In response to this concern, specific AF stewardship programmes (AFS) are being developed to improve the clinical outcome of patients receiving AF drugs and to ensure optimal prescription in terms of spectrum, dose, therapeutic drug monitoring, duration and route of administration while avoiding adverse effects and unnecessary costs [3]. Interventions are extrapolated from the antibiotic stewardship programmes and include audits with feedback, guideline and protocol development, educational programmes and prescription evaluation [4, 5]. Although AFS are relatively new, a systematic review in 2017 identified 14 papers describing AFS in adult patients and the improvements in AF prescription and use after implementation [6]. In contrast, data about paediatric AFS are scant and mainly limited to adult programmes including children or to specific situations such as invasive *Candida* spp. infection or cancer [7, 8].

The aim of this PROAFUNGI study was to describe the use and appropriateness of AF in a high complexity paediatric centre.

## Methods

The children’s hospital at Vall d’Hebron Barcelona Hospital Campus is a reference tertiary care centre with a national reference programme of solid organ transplantation (SOT) and hematopoietic stem cell transplantation (HSCT) with 35 and 40 yearly transplantations, respectively, as well as a 20-bed paediatric ICU (PICU), a 44-bed neonatal ICU (NICU) and a large surgery programme with around 2700 inpatient procedures per year. The institutional paediatric antimicrobial stewardship program (PROA-NEN) was formally founded in 2015 and aims to improve clinical outcomes, reduce antimicrobial-related adverse events and ensure the cost-effective use of treatments. During 2018, a specific AFS team was created, involving 3 pharmacists, 3 paediatric infectious diseases specialists and 1 microbiologist.

The PROAFUNGI study is an observational, prospective, single-centre, modified point-prevalence survey (mPPS) study performed from July to October 2018. Admitted patients receiving AF were identified through the electronic prescription system (Silicon® v11, Grifols International, S.A., Sant Cugat del Vallès, Spain and Centricity™ Critical Care 8.1 SP7, General Electric Company, Barrington, United States) and data were collected weekly. All consecutive paediatric (< 18 years) patients receiving at least one systemic AF were included. Patients were included again if a new AF course was started. Additionally, one AF prescription was recorded for each week in which the drug was prescribed. Patients receiving AF for less than 48 h were excluded.

Demographic, clinical and treatment data were obtained from the patient’s electronic medical record (SAP© NetWeaver 7.0 SPS37, California, United States), including age, sex, underlying disease, AF indication and IFI category. The patient’s IFI was classified as possible, probable or proven according to the European Organization for Research and Treatment of Cancer/Mycoses Study Group (EORTC/MSG) definitions [9]. Additionally, patients at risk of IFI and or with clinical suspicion of IFI were added as other categories to include situations not listed in the EORTC/MSG criteria. The AF indication was classified as prophylaxis (primary or secondary) or treatment (empirical, pre-emptive or targeted). Pharmacological data (AF, route of administration, dose and frequency) were collected from the abovementioned electronic prescription systems.

After the collection, data were evaluated by two independent members of the AFS team. In case of disagreement, a third member was consulted to achieve consensus. Following the guidelines for the implementation of antimicrobial stewardship programs in Spanish hospitals, optimal prescription was defined as “necessary” (if the clinical situation required the use of an AF, either prophylactic and therapeutic), “appropriate” (if the drug was active against the causative microorganism and adjusted to the patient’s characteristics), and “adequate” (if the dose, duration and route of administration were correct, while in concordance with local protocols, or international guidelines if local protocols were not available) [10].

Qualitative variables were described as the number and percentage, and quantitative variables as the mean ± standard deviation (SD) or median (IQR). The statistical analysis was carried out with Microsoft® Office Excel® 2007 (12.0.4518.1014).

The local Ethics Committee for Clinical Research approved the study in March 2018 (code VAL-ANT-2017-01).

## Results

### Patient and fungal infection characteristics

During the study period, 55 patients were included; 53% were males and median age was 8.7 years (IQR 2.4–13.8 years). The main underlying condition was cancer (25, 45.5%; 15 [60%] of them having undergone HSCT); and the most common situation leading to AF prescription was patients at risk of IFI (27; 49%). Detailed data are provided in Table 1.

**Table 1:** Patient Characteristics

	Total
Clinical characteristics, *n*	55
• Age, y, median (IQR)	8.7 (2.4–13.8)
• Male sex, *n* (%)	29 (53)
Underlying disease	
• Cancer, *n* (%)	25 (45.5)
• *HSCT*, *n* (%)	*15/25 (60)*
• Critical paediatric patients, *n* (%)	14 (25.5)
• Critical newborns, *n* (%)	5 (9)
• Primary immunodeficiencies, *n* (%)	5 (9)
• Solid organ transplant, *n* (%)	3 (5.5)
• Other, *n* (%)	3 (5.5)
Clinical situation^a^	
• Risk of IFI, *n* (%)	27 (49)
• Suspicion of IFI, *n* (%)	6 (11)
• Possible IFI, *n* (%)	14 (25)
• Probable IFI, *n* (%)	2 (4)
• Proven IFI, *n* (%)	6 (11)
Microorganisms	
• *Candida* spp.	2/6
• Moulds	4/6

Abbreviations: *IFI* invasive fungal infection

^a^IFI category was defined according to the European Organization for Research and Treatment of Cancer/Mycoses Study Group (EORTC/MSG) definitions

### Antifungal prescription characteristics

A total of 119 AF prescriptions were analysed. The indication was prophylaxis in 75 (63.2%) cases (70 primary and 5 secondary) and treatment in 44 (36.8%): 30 (25%) empirical; 1 (0.8%) pre-emptive; 13 (11%) targeted. Drug distribution by type of patient and indication is detailed in Table 2. Most drugs (93.3%) were prescribed as monotherapy (52 patients), while dual and triple therapy were only used in 1 patient (2 prescriptions, 1.7%) and 2 patients (6 prescriptions, 5%), respectively.

**Table 2:** Antifungal Prescription Characteristics and Adequacy

		Total	Prophylaxis	Treatment
**Antifungal prescriptions,** ***n***		119	75	44
Indication for AF therapy				
Primary prophylaxis, *n* (%)		70 (58.8)	70 (93.3)	
Secondary prophylaxis, *n* (%)		5 (4.4)	5 (6.7)	
Empirical therapy, *n* (%)		30 (25)		30 (68.2)
Pre-emptive therapy, *n* (%)		1 (0.8)		1 (2.3)
Targeted therapy, *n* (%)		13 (11)		13 (29.5)
Underlying disease				
Cancer, *n* (%)		64 (54)	48 (64)	16 (36.4)
* HSCT*, *n* (%)		*43/64 (67)*	*35/48 (73)*	*8/16 (50)*
Critical paediatric patients, *n* (%)		29 (24)	16 (21.4)	13 (29.5)
Critical newborns, *n* (%)		7 (6)	3 (4)	4 (9.1)
Primary immunodeficiencies, *n* (%)		11 (9)	7 (9.3)	4 (9.1)
Solid organ transplant, *n* (%)		3 (3)	0 (0)	3 (6.8)
Other, *n* (%)		5 (4)	1 (1.3)	4 (9.1)
AF agent				
Liposomal amphotericin B, *n* (%)		55 (46.2)	41 (54.6)	14 (32)
Posaconazole, *n* (%)		21 (17.7)	21 (28)	0 (0)
Anidulafungin, *n* (%)		13 (10.9)	3 (4)	10 (23)
Fluconazole, *n* (%)		12 (10.1)	6 (8)	6 (14)
Micafungin, *n* (%)		6 (5.0)	2 (2.7)	4 (9)
Isavuconazole, *n* (%)		5 (4.2)	0 (0)	5 (11)
Voriconazole, *n* (%)		4 (3.4)	0 (0)	4 (9)
Itraconazole, *n* (%)		2 (1.7)	2 (2.7)	0 (0)
Caspofungin, *n* (%)		1 (0.8)	0 (0)	1 (2)
**Antifungal prescription evaluations,** ***n***		219	144	75
Non-optimal prescriptions, *n* (%)		24 (11)	12 (8)	12 (16)
Unnecessary, *n* (%)		14 (6)	10 (7)	4 (5)
Inappropriate, *n* (%)		2 (1)	0 (0)	2 (3)
Inadequate	Incorrect dose, *n* (%)	7 (3)	4 (3)	3 (4)
	Incorrect route of administration, *n* (%)	0 (0)	0 (0)	0 (0)
	Incorrect duration, *n* (%)	7 (3)	7 (5)	0 (0)
	Disagreement with institutional protocol, when available, *n* (%)	12 (8)^a^	4 (3)^a^	8 (20)^a^
Non-optimal prescription distribution among underlying disease, *n* (%)				
Cancer		4 (16.7)	2 (16.7)	2 (16.7)
* HSCT*, *n* (%)		*4/4 (100)*	*2/2 (100)*	*2/2 (100)*
Critical paediatric patients, *n* (%)		14 (58.3)	8 (66.7)	6 (50)
Critical newborns, *n* (%)		1 (4.2)	1 (8.3)	0 (0)
Primary immunodeficiencies, *n* (%)		1 (4.2)	1 (8.3)	0 (0)
Solid organ transplant, *n* (%)		1 (4.2)	0 (0)	1 (8.3)
Other, *n* (%)		3 (12.5)	0 (0)	3 (25)

Abbreviations: *AF* antifungal

^a^ Calculated as a percentage of the prescriptions for which a local institutional protocol was available: 157 total, 117 prophylaxis, 40 treatment

### Prescription quality assessment

During the study period, the PROAFUNGI working group performed a total of 11 surveys, including 219 prescription evaluations. From the 219 evaluated prescriptions, 24 (11%) were considered non-optimal. As shown in Table 2, the main reason for a non-optimal evaluation was lack of indication (14/24), followed by disagreement with the local protocols (12/24), mainly in heart-transplanted and HSCT patients. Institutional protocols were available for 117/144 (81%) prophylactic and 40/75 (53%) treatment prescriptions. Moreover, the departments with the highest AF use (cancer department and PICU) issued most non-optimal prescriptions (16.7 and 58.3%, respectively).

Liposomal amphotericin B (LAMB) was used commonly and was also the main drug considered non-optimal (Table 3), essentially due to non-optimal prescriptions related to prophylaxis in patients receiving circulatory assistance (7/12): 2 patients with ventricular assist device (VAD) who had an unnecessary prescription and 1 receiving extracorporeal membrane oxygenation (ECMO) support who had an inappropriate prescription due to an excessive dosage.

**Table 3:** Assessment of Prescription Quality According to Drug

Drug	Patients, *n*	Prescriptions Evaluated, *n*	Non-optimal Prescriptions (*n*, %)	Cause ^a^
Liposomal amphotericin B	27	105	12 (11%)	- Unnecessary (9)
				- Inappropriate (2)
				- Inadequate: incorrect dose (5), incorrect duration (7), disagreement with institutional protocol ^b^ (5)
Posaconazole	12	32	1 (3%)	- Inadequate, incorrect dose (1)
Anidulafungin	11	29	3 (10%)	- Unnecessary (3)
Fluconazole	11	25	1 (4%)	- Inadequate, incorrect dose (1)
Isavuconazole	2	9	5 (56%)	- Inadequate: disagreement with institutional protocol** (5)
Micafungin	3	7	2 (29%)	- Unnecessary (2)
				- Inadequate: disagreement with institutional protocol ^b^ (2)
Voriconazole	2	5	0	N/A
Caspofungin	1	4	0	N/A
Itraconazole	2	3	0	N/A

Abbreviations: *N/A* not applicable

^a^ The same prescription may not be optimal for more than one reason

^b^ Disagreement with institutional protocol, when available

Two non-approved drugs in paediatrics at the time of the study, isavuconazole (5 prescriptions in one patient) and anidulafungin (3 prescriptions in one patient), accounted for a significant number of non-optimal prescriptions due to the availability of alternative approved drugs as empirical treatment in one patient with HSCT and acute renal impairment who could have been treated with oral voriconazole and one patient with portal cavernomatosis who could have been treated with micafungin, respectively.

## Discussion

The present study demonstrates a significantly high percentage of optimal prescription of AF in children at a referral hospital but has also detected some points of improvement mainly related to prophylaxis in critically ill patients receiving circulatory assistance and the use of non-approved drugs in children.

According to previous published articles, cancer patients were those receiving AF more frequently [8, 11, 12]. In a retrospective cohort study conducted in 25 paediatric hospitals in the USA, patients with malignancies represented 42% of overall AF use [11]. In our cohort, a large proportion of AF prescriptions were ordered for HSCT patients (15 patients, accounting for 43 prescriptions, 36% of all AF prescriptions and 67% of cancer patient prescriptions), as our centre is a national referral transplant unit. Similarly, in a study including only children with cancer, HSCT patients accounted for 78% of overall AF prescriptions [8]. Regarding patients’ age, the Antibiotic Resistance and Prescribing in European Children (ARPEC) study reported higher AF use in paediatric patients (83%) compared with newborns (17%), as did another survey including more than 50 hospitals in the USA (76 DOT/1000 patient-days in paediatric patients compared with 14 DOT/1000 patient-days in newborns) [12, 13]. Remarkably, AF use in neonates was even lower in our centre (5 patients, accounting for 7 prescriptions, 6%), in relation to a minimum incidence of neonatal candidiasis (0.6%) that precludes the use of universal AF prophylaxis in low birth weight neonates [14, 15].

Regarding AF indication, prophylaxis was the main reason for prescriptions (63.2%), followed by empirical treatment (25%) and targeted treatment (11%). These findings are consistent with data from the study performed by Santiago-García et al. that included 56 children with cancer and also the arm of paediatric patients from the ARPEC study [8, 12]. In the neonate arm, prophylaxis was the reason for 46% of prescriptions, while treatment of suspected candidaemia accounted for the other 44% [12].

In terms of the distribution of AF, LAMB was the most prescribed drug in our study, followed by posaconazole. At our hospital, low-dose LAMB is the drug of choice for AF prophylaxis in high-risk cancer patients (relapsed or refractory leukaemia, HSCT pre-engraftment phase) due to its effectiveness and safety profile as shown in a previous paper by our group [16]. LAMB is also used in critically ill paediatric patients due to its stability with the concomitant use of continuous renal replacement therapy, as opposed to azole elimination [17]. On the other hand, posaconazole is mainly indicated in the HSCT post-engraftment period and in some primary immunodeficiencies (PID) such as chronic granulomatous disease. These data was quite different from other studies in which fluconazole is the most commonly prescribed agent [7, 11–13]. Nonetheless, all these studies include a high proportion, or exclusively include, neonates in whom *Candida* spp. is the main and almost only fungal pathogen [7, 11, 12]. Conversely, for paediatric, particularly children with cancer, PID or HSCT (a majority in our cohort), filamentous fungi should be considered, and other AFs with a broader spectrum play a major role [2, 10, 18, 19].

Regarding AF prescription evaluations, a high proportion (89%) of prescriptions were considered correct, similar to the study by Santiago-García et al., that described 93% of appropriate AF prescriptions in a paediatric cancer department [8]. In contrast, a multicentric study by Ferreras-Antolín et al. considered that only 34% of prescriptions were optimal, similar to another study focused on adult patients with hospital-acquired candidemia that found 47% of correct prescriptions [7, 20]. Remarkably, the level of non-optimal prescriptions was higher in patients receiving treatment (12/75; 16%) compared with prophylaxis (12/144, 8%), probably due to the higher availability of institutional protocols aimed at prophylaxis. Protocols were available for 117/144 (81%) prophylactic prescriptions and for 40/75 (53%) treatment prescriptions.

Regarding the cause of incorrect prescription, the leading reason was the lack of indication (unnecessary) (14/24), followed by disagreement with current recommendations (12/24), incorrect dose (7/24), incorrect duration (7/24) and inappropriate choice of AF (2/24). Interestingly, the proportions were generally similar to Santiago-Garcia et al., that described the lack of indication as the main cause of non-optimal prescription (7/13), followed by incorrect dosage (5/13) or incorrect route of administration (1/13) [8].

Some incorrect prescriptions were related to drugs not approved for paediatric use at the time of study (such as anidulafungin or isavuconazole). Although these drugs have shown clinical benefits in adults, paediatric use should be carefully evaluated and restricted to cases with no approved alternative for children. Moreover, for improving prescription’s quality, decisions regarding antifungals should be made by a multi-disciplinary team. In centres with persuasive ASP, following experts’ advice is of utmost importance.

With regards to dose, the incorrect rate (3%) was significantly lower than in a previous study by Lestner et al. that found an inadequate dose as the main cause of incorrect AF use in paediatrics, identifying 47% of cases with doses lower than recommended by international guidelines [12]. The existence of specific tools for electronic prescription and the daily revision by the Pharmacy Department have probably contributed to this high rate of adequate dosing.

The limitations of this study were its single-centre nature and its PPS format, which may have meant that some prescriptions were missed. Moreover, a 4-month period including 11 surveys may not be sufficiently representative of overall practice, as several periods of viral epidemics can produce fever without bacterial cause and thus lead to increased empirical AF use. However, the results of this study regarding AF use are similar to annual AF consumption (DOT/100 PD) in our hospital (unpublished data). In 2018, most AF usage occurred in standard hospitalization patients, followed by PICU patients (63 and 34%, respectively). In our cohort, these patients accounted for 70 and 24% of prescriptions, respectively. By therapeutic group, the AFs used most often in 2018 were azoles (45% DOT/100 PD), LAMB (32% DOT/100 PD), and echinocandins (23% DOT/100 PD). In terms of AF distribution in this PROAFUNGI study; LAMB was the most prescribed drug (46.2%), followed by azoles (37%) and echinocandins (16.7%).

## Conclusions

A high rate of optimal AF evaluation was globally demonstrated for all areas of a tertiary children’s hospital, in relation to a well-established ASP that performs weekly audits and agreed protocols. However, this study identified specific areas of improvement, most of them related to the lack of an institutional protocol. These findings will guide the next actions of the AFS team, which will focus mainly on critical patients and the use of non-approved drugs in children.

## Data Availability

The datasets used and/or analysed during the current study are available from the corresponding author on reasonable request.

## Abbreviations

AF: Antifungal drugs
AFS: Antifungal stewardship programmes
ARPEC: Antibiotic Resistance and Prescribing in European Children
ECMO: Extracorporeal membrane oxygenation
EORTC/MSG: European Organization for Research and Treatment of Cancer/Mycoses Study Group
HSCT: Hematopoietic stem cell transplantation
IFI: Invasive fungal infection
LAMB: Liposomal amphotericin B
mPPS: modified point-prevalence survey
NICU: Neonatal ICU
PICU: Paediatric ICU
PID: Primary immunodeficiencies
SOT: Solid organ transplantation
VAD: Ventricular assist device
